# Influence on Consultation Behavior of Pregnant and Postpartum Women in Japan: Insights from a Nation-Wide Survey

**DOI:** 10.3390/healthcare13121422

**Published:** 2025-06-13

**Authors:** Yuri Kita, Teruhide Koyama, Takahiro Tabuchi, Miho Shizawa

**Affiliations:** 1 Graduate School of Nursing for Health Care Science, Kyoto Prefectural University of Medicine, Kyoto 602-8566, Japan; 2 Department of Epidemiology for Community Health and Medicine, Kyoto Prefectural University of Medicine, Kyoto 602-0841, Japan; 3 Faculty of Regional Promotion, Nara Prefectural University, Nara 630-8258, Japan; 4 Division of Epidemiology, School of Public Health, Tohoku University Graduate School of Medicine, Sendai 980-8574, Japan

**Keywords:** receiving support, consultation behavior, pregnant women, postpartum women, primigravidae

## Abstract

**Background**: In Japan, new challenges are emerging, such as declining birth rates, an increase in age at childbirth, postpartum depression, and child abuse. **Methods**: This study examines the factors influencing maternal consultation behavior at public institutions using some of the data from the Japan COVID-19 and Society Internet Survey (JACSIS). A total of 6227 women (1380 pregnant and 4847 postpartum) were analyzed through logistic regression. **Results**: The results showed different factors influencing consultations between pregnant women and postpartum women. Among pregnant women, different trends were observed between primigravidae and multigravidae, indicating that primigravidae aged ≥40 years and multigravidae with multiple childbirths tend to seek consultations more frequently. In postpartum women, psychological distress (K6), adverse childhood experiences (ACEs), and social support networks significantly influenced consultation behavior. Postpartum women with extensive support networks (≥3 confidants) exhibited higher consultation rates. In contrast, those with elevated Edinburgh Postnatal Depression Scale (EPDS) and Mother-to-Infant Bonding Scale (MIBS-J) scores were less likely to seek help, potentially due to mental health stigma. **Conclusions**: This study showed that pregnant and postpartum women facing physical, mental, family, or relationship issues are seeking consultations and receiving thorough support. On the other hand, since some pregnant and postpartum women refrained from seeking consultation, it is necessary to further consider support measures that make it easier for all pregnant and postpartum women to seek advice in the future.

## 1. Introduction

In 2023, the total fertility rate dropped to 1.20 per woman from the previous year’s 1.26, marking a record low in Japan [1]. The birth rate is declining, but families and communities cannot raise children comfortably [2]. A prime example is child abuse. In Japan, there has been a notable increase in the reported number of child abuse cases, escalating from 11,631 in 1999 to 219,170 in 2022 [1]. Child abuse is associated with perinatal depression in women. The prevalence of perinatal depression among Japanese women is high, ranging from 11.5% to 15.1% [3]. A previous study on postpartum depression found that both a woman’s perception of mental illness and a lack of social support before pregnancy are associated with postpartum depression [4]. Also, it has been revealed that adverse childhood experiences strongly influence maltreatment between mother and child after birth [5]. Edinburgh Postnatal Depression Scale (EPDS) scores, used as a screening tool for perinatal depression, tend to increase during early pregnancy and one month postpartum, approaching the cutoff point [6]. Previous research has shown that during pregnancy and postpartum, women go through a process of recognizing their challenges and seeking support [7]. Women in the perinatal period experience various concerns at different stages of pregnancy, childbirth, and childrearing, and their situations change over time. In general, pregnancy is considered to involve more worries, anxieties, and physical discomforts than usual. Especially for pregnant women who have no one to consult or support them, even if supporters recognize the need to provide assistance, the pregnant women themselves often do not feel troubled and do not seek help [8]. In contrast, between 50 and 60% of postpartum women reported having difficulties during the period from discharge from the maternity facility to one year postpartum. Additionally, 80–90% of respondents cited “family support,” primarily from their husbands and mothers, in addressing these difficulties. About 20–30% of respondents dealt with these issues with the support of professionals [9]. In the context of nuclear families, the ratio of unpaid labor time (housework and childcare) between Japanese women and men is 5.5, which is a larger gender gap compared to other countries, placing a heavier burden on women [10]. Other issues faced by perinatal women include weakening family functions [11], lack of support, difficulties in interacting with children, and financial problems [12]. During the perinatal period, the background factors that lead pregnant and postpartum women to seek support and consult professionals differ. Although previous studies have focused on the characteristics of primiparous and multiparous women in the postpartum period, few studies have examined the characteristics of primigravidae and multigravidae during pregnancy or explored the appropriate forms of support for each group.

Since the enforcement of the Maternal and Child Health Act, Japan’s maternal and child health care system has been systematized and improved. In Japan, a medical doctor confirms that a woman is pregnant at a medical institution. She notifies the local government of her pregnancy, receives a Maternal and Child Health Handbook (MCH handbook), and undergoes continuous antenatal checkups at medical institutions until delivery. Local governments provide home visits during the pregnancy and postpartum period, mainly by public health nurses, who are responsible for community health. After childbirth, children undergo infant and early childhood health examinations (during infancy, at ages 1.5 and 3, etc.) at the municipality. The Children and Families Center is one of the key institutions responsible for maternal and child health activities. In 2024, under the Child and Family Agency, Child and Family Centers were established in all municipalities across Japan to build a comprehensive support system. These centers serve as central institutions providing support through both population-based and high-risk approaches [13]. There are also reports that public health nurses tend to focus on continuous involvement with high-risk cases and hesitate to actively intervene with individuals who are not considered high-risk [14].

In recent years, the concept of “receiving support” has gained attention in Japan. In 2012, the Cabinet Office (in charge of disaster prevention) introduced the term “receiving support” as a “keyword for accepting volunteers in the community” [15]. It is necessary not only to focus on the activities of disaster relief volunteers but also to improve their ability to make the most of the support they receive. If the balance of these forces is unbalanced, it can lead to complacency and dependency [16]. From a healthcare behavioral science perspective, receiving support is defined as the ability of individuals with health problems to accept and utilize support to address their problems [17]. Summarizing prior research, this concept underscores the importance of encouraging women to seek support independently, fostering an attitude of receiving and utilizing support for maternal mental health, and providing support aligned with women’s needs, considering familiar supporters and the childcare environment [18,19]. This study aims to clarify the factors that influence “receiving support” among pregnant and childbearing mothers, with consulting public institutions defined as a manifestation of receiving support. There are hardly any studies that identify the factors connecting perinatal women to counseling services. This study aims to clarify the underlying factors that led pregnant and postpartum women to seek consultations with public institutions. The significance of the research lies in identifying the factors that influence the consultation behaviors of these women, providing foundational data to explore ways to support those who proactively seek consultations as well as those who do not, ensuring appropriate assistance is offered as needed.

## 2. Materials and Methods

### 2.1. Definitions

#### Consultation Behavior

From the perspective of the behavioral sciences of health care, “receiving support” is the ability of an individual with a health problem to accept and use support to solve the problem [17].

We defined consultation behavior as seeking advice at public service facilities (such as government offices, health centers, or childrearing support centers) or medical institutions.

### 2.2. Study Design and Participants

This study was a cross-sectional study.

Data were collected through the Japan COVID-19 and Society Internet Survey (JACSIS) Research Expectant Mothers, Partners, and Childrearing Generation Survey, an online survey conducted across Japan. The survey was administered from 24 July to 30 August 2021 to panel members of an Internet research firm, covering 14,086 pregnant and postpartum women (11,661 postpartum and 2425 during pregnancy). Responses were obtained from 8047 pregnant and postpartum women (response rate of 57.1%) among the target women. After excluding inconsistent answers (n = 721), 7326 respondents remained, of whom 6227 (4847 postpartum and 1380 pregnant) were included in the analysis, excluding those who did not provide information on annual income. In this study, we utilized part of the data obtained and analyzed data related to consultations from pregnant and postpartum women, excluding data concerning COVID-19. We did not set any exclusion criteria.

### 2.3. Measure of Outcome Variables

#### 2.3.1. Basic Information

Responses related to age, highest education level attained, employment status, income, and partner’s income were obtained. Age was divided into three deciles. Education level was classified into high school graduates or less and university graduates or more. Employment status was divided into “employed” (executives, self-employed, full-time employees, temporary workers, contract workers, part-time workers, and in-house workers) and “not employed” (students and others). Income was also divided into three deciles.

#### 2.3.2. Physical and Obstetrical Information

Responses related to sleep duration (hours), number of births, method of delivery (vaginal or cesarean), week of delivery, baby’s weight (g), and pregnancy-related abnormalities were obtained. Abnormality was defined as answering “yes” to one or more of the thirteen postpartum abnormalities listed.

#### 2.3.3. Mental Health Information

K6 Score:

This is a six-item scale developed by Kessler et al. for screening mental disorders such as depression and anxiety. Higher scores indicate greater psychological distress [20].

Edinburgh Postnatal Depression Scale (EPDS):

This is a ten-item scale to screen postpartum depression, with a Japanese cutoff point of 9. Scores of 9 or higher suggest likely depression [21,22,23].

Mother-to-Infant Bonding Scale (MIBS-J):

This is a ten-item scale assessing emotional bonds between mothers and children, categorized into Lack of Affection (LA) and Anger/Rejection (AR). Higher scores indicate stronger negative feelings toward the child [24,25].

Happiness Score:

This is a self-reported scale from 1 (not happy) to 10 (very happy).

Communicative and Critical Health Literacy (CCHL):

This is a scale measuring functional, interactive, and critical health literacy. Higher average scores indicate better health literacy [26,27].

Description of problems:

Respondents also answered questions on problems, worries, or anxieties, selecting “yes” or “no” for all seven items.

#### 2.3.4. Household and Relationship Information

We collected data on household and relationship factors, including the number of people in the household, the daily time spent on housework and childcare, and the number of individuals the respondent could consult. The number of household members refers to the family members living together. The time spent on housework and childcare is recorded as the average daily duration. The number of people available for consultation is categorized into three groups.

Family APGAR: (Adaptability, Partnership, Growth, Affection, and Resolve (APGAR), hereafter referred to as FA):

This measure includes five questions designed to assist physicians in assessing family functionality within clinical practice. A total FA score of 7 to 10 indicates a highly functional family, a score of 4 to 6 suggests moderate dysfunction, and a score of 0 to 3 signifies severe dysfunction [28]. Another study reports that scores ranging from 5 to 10 reflect high levels of dysfunction [29].

ACEs (adverse childhood experiences, hereafter referred to as ACEs):

Epidemiological studies have demonstrated that ACEs impact mental and physical health into adulthood and beyond [30]. Exposure to ACEs increased the risk of pregnancy complications and adverse pregnancy outcomes [31]. Respondents were asked to respond “Yes” or “No” to all 10 listed ACEs.

### 2.4. Statistical Analysis

Since the factors leading to consultation behavior were considered to differ between pregnant women and postpartum women, the subjects were divided into these two groups for analysis. Additionally, for pregnant women, the risk of “advanced maternal age at first birth” was taken into account as it could affect the results [32], and a sub-analysis was conducted focusing only on primigravidae (Supplementary Materials). Descriptive statistics were computed for all survey items.

Pregnant and postpartum women were further categorized based on their responses regarding whether they had consultations. Those who selected “Yes, I consulted” were classified as the consultation group, while those who selected either “No, I did not consult” or “I wanted to consult but did not” were classified as the non-consultation group. Chi-square tests were conducted to compare categorical variables between the two groups, while *t*-tests or Mann–Whitney U-tests were performed for continuous variables.

Continuous variables were tested for normal distribution. For variables such as sleep duration, weeks of delivery, birth weight, and CCHL, *t*-tests were applied. For non-normally distributed variables, including K6, EPDS, MIBS-J, happiness scores, household time, and Family APGAR, Mann–Whitney U-tests were used.

A forced-entry binary logistic regression analysis was conducted to identify factors related to consultation status for both pregnant and postpartum women. The dependent variable was consultation status (consulted or not), and all survey items were used as independent variables. For independent variables, the continuous variables are sleep duration, number of births, gestational age of childbirth, birth weight, K6 Scale, EPDS, Bonding Scale, happiness, CCHL, number of household members, time spent on housework, and Family APGAR. The categorical variables are age (<34, 34–39, 40≤), annual income (JPY <1.3 million, 1.3–3.0, 3.0<), partner’s annual income (JPY <1.3 million, 1.3–5.0, 5.0<), education (college or higher, high school graduate or lower), occupational status (employed, unemployed), abnormalities during pregnancy (no, yes), postpartum anomalies (no, yes), problems (no, yes; pregnancy and childbirth, economic, physical, marital relationship, family relationships, childcare and children, work), the number of persons available for consultation (≤2, 3–10, 11≤), and ACEs (no, yes; parent’s death, divorced parents, parental mental illness, violence from father to mother, physical abuse, neglect, emotional abuse, economic poverty, bullying, sexual victimization). Categorical variable contrast was the first category.

The statistical software IBM SPSS Statistics version 27 was used for the analysis, and odds ratios (ORs) and 95% confidence intervals were calculated, with statistical significance set at *p* < 0.05.

## 3. Ethical Procedure

This study complies with the Declaration of Helsinki of 1975 and the Code of Ethics revised in 2008 and was approved by the Osaka International Cancer Center Research Ethics Committee (approval date 19 June 2020, approval number 20084). Data were provided by the research company in compliance with the Personal Information Protection Law. In addition, approval from the Ethics Committee of Kyoto Prefectural University of Medicine was obtained for the analysis (approval no. ERB-C-2087-2).

## 4. Result

### 4.1. Characteristics of the Pregnant Women

Of the 1380 pregnant women, the group that received counseling reported having shorter sleep duration times and significantly more childbirth experiences. Mental health scores, including K6 and CCHL, were significantly higher in the consultation group. Physical problems, childrearing issues, and work-related concerns were more prevalent in the consultation group. Household size, time spent on housework and childcare, and Family APGAR scores were also higher. Significant differences in ACEs (e.g., physical/emotional abuse and bullying) were observed between the groups.

Among pregnant women, differences were observed between the consultation group and the non-consultation group in the number of births, time spent on housework and childcare, and issues related to childrearing, which were indicated as characteristics of multigravidae (Table 1).

### 4.2. Characteristics of Postpartum Women

Among 4847 postpartum women, the consultation group had a higher proportion of employed individuals and reported more postpartum abnormalities. Higher K6, happiness, and CCHL scores were observed. Issues such as financial concerns, family/marital relationships, and childrearing were more common. Differences were also observed between the groups in the number of people available for consultation and ACEs such as parental mental illness and bullying.

Since no difference was observed between the consultation group and the non-consultation group in the variable of the number of childbirths among postpartum women, the analysis was conducted without separating primiparous and multiparous participants (Table 2).

### 4.3. Results of Logistic Regression Analysis of Pregnant and Postpartum Women Who Consulted Public Institutions

Table 3 shows the results of the binary logistic regression analysis conducted to identify the factors influencing whether pregnant and postpartum women consulted institutions.

#### 4.3.1. Result of Pregnant Women

For pregnant women, a higher frequency of previous births was associated with an increased likelihood of consultation, with an OR of 1.40 (95% confidence interval [CI]: 1.11–1.77). Additionally, those who reported concerns regarding children and childcare were more likely to seek a consultation, with an OR of 1.81 (95% CI: 1.37–2.39). Furthermore, the time spent on housework and childcare had an odds ratio of 1.04 (95% CI: 1.01–1.07). Experiences of bullying during childhood or adolescence, as measured by the ACE questionnaire, were also positively associated with consultation behavior, with an OR of 1.42 (95% CI: 1.07–1.88).

#### 4.3.2. Result of Postpartum Women

For postpartum women-related factors, educational attainment was a significant predictor, with individuals who completed high school or less being more likely to seek a consultation compared to those with higher education (OR: 1.31, 95% CI: 1.15–1.48). Regarding age, women aged 34 to 39 were more likely to seek a consultation compared to women under 34 (OR: 1.15, 95% CI: 1.01–1.31). Postpartum anomalies were also positively associated with consultation behavior, with an OR of 1.23 (95% CI: 1.04–1.45). Higher scores on the K6 scale, which measures psychological distress, were associated with an increased likelihood of consultation (OR: 1.02, 95% CI: 1.01–1.03). Similarly, higher scores on happiness and CCHL (health literacy) were positively associated with consultation, with ORs of 1.09 (95% CI: 1.04–1.14) and 1.05 (95% CI: 1.03–1.06), respectively.

Consultation behavior was also influenced by specific difficulties. Respondents who reported physical difficulties, childcare concerns, or work-related issues were more likely to seek a consultation, with an OR of 1.28 (95% CI: 1.12–1.47), 1.62 (95% CI: 1.42–1.86), and 1.19 (95% CI: 1.04–1.37), respectively. Furthermore, the number of persons available for consult played a significant role. Respondents with access to three to ten persons available for consult had an OR of 1.58 (95% CI: 1.39–1.80), while those with access to eleven or more persons available for consult exhibited an even higher likelihood of consultation, with an OR of 2.08 (95% CI: 1.22–3.55).

Adverse childhood experiences also influenced consultation behavior. Respondents who reported parental mental illness as an ACE were more likely to seek a consultation, with an OR of 1.61 (95% CI: 1.23–2.11), as were those who reported bullying, with an OR of 1.26 (95% CI: 1.09–1.46). In contrast, higher scores on certain psychological measures reduced the likelihood of consultation. Specifically, higher scores on the EPDS (Edinburgh Postnatal Depression Scale) and MIBS-J (Mother-to-Infant Bonding Scale) were associated with decreased consultation behavior, with ORs of 0.98 (95% CI: 0.96–1.00) and 0.98 (95% CI: 0.96–1.00), respectively.

### 4.4. Results of Logistic Regression Analysis of Primigravidae Who Consulted Public Institutions

Table 4 presents the results of a binomial logistic regression analysis of factors affecting whether primigravidae consulted institutions.

For primigravidae, advanced maternal age (40≤) was associated with a higher likelihood of consultation compared to women aged <34, with an OR of 2.94 (95% CI: 1.19–7.28). Greater time spent on housework and childcare was also a significant predictor of consultation, with an OR of 1.80 (95% CI: 1.04–3.11). Emotional abuse and bullying, as reported in the ACE questionnaire, were positively associated with consultation, with ORs of 1.81 (95% CI: 1.05–3.12) for emotional abuse and 1.54 (95% CI: 1.03–2.30) for bullying.

## 5. Discussion

The pregnant and postpartum women included as participants in this study were generally more highly educated than the average Japanese female population but otherwise shared similar demographic characteristics with the general population.

In the logistic regression analysis of factors influencing consultation behavior, pregnant and postpartum women who had risks related to physical, mental, family, and interpersonal matters were more likely to seek a consultation, with distinct trends and characteristics observed among primigravidae.

### 5.1. Pregnant Women

Among pregnant women, the odds ratio (OR) was 1.40 for the number of births and 1.81 for problems related to children and childcare. Based on these results, the characteristics of multigravidae—women defined as being pregnant with their second or subsequent child—were observed. Families of multigravida women face the developmental task of integrating a new family member (the unborn child) while addressing the needs of their older child [33]. These women and their families may experience confusion and anxiety during this transitional period. Prenatal checkups offer opportunities for multigravidae to consult professionals, such as addressing concerns about their relationship with their firstborn. Furthermore, support is available through children’s health checkups, pediatric consultations, and childcare facilities such as nurseries and daycare centers. Multigravidae likely utilized these various opportunities to seek assistance regarding childrearing and childcare-related issues. Additionally, among pregnant women overall, there was a slight tendency to engage in consultation behavior related to the amount of time spent on housework and childcare, with an OR of 1.04. Among them, it has been reported that multigravidae who have had one child experience more fatigue than those who have had two or more children [34], so it is important to pay attention to the physical and mental strain related to childcare in these women. Furthermore, in primigravidae, an OR of 1.80 for time spent on housework and childcare was observed to affect consultations. This may be due to the physical changes and discomfort experienced during the first pregnancy, which can be affected by the household burden. Support from close family and friends is essential, and if such support is not available, it is necessary to seek social assistance. Previous studies have observed that social support correlates positively with the QoL in all its dimensions. This implies that the social support of pregnant women acts as a protective factor for good QoL [35]. Therefore, for pregnant women, it is necessary to assess their household and childcare situations and provide support accordingly.

In the sub-analysis on primigravidae, those aged 40 and above (OR 2.94) and those with experiences of psychological abuse (OR 1.81) were significantly more likely to seek consultation. In Japan, the proportion of primigravidae at an advanced age is increasing, leading to a rise in postpartum hemorrhage cases [36]. Studies in high-income countries have consistently shown a correlation between advanced maternal age and heightened risks of adverse birth and maternal outcomes. Individualized, patient-centered interventions are essential throughout pregnancy [37,38]. In Japan, primigravidae aged 40 and above are classified as “high-risk pregnancies” and are closely monitored and supported. Also, primigravidae who report experiencing psychological abuse when registering their pregnancy are designated as “Specified Expectant Mother (as defined under the Child Welfare Act of Japan” or “pregnant women requiring support” and receive focused assistance from healthcare professionals. The results confirm the necessity of the current extensive support for high-risk primigravidae.

Regarding educational background, having a high school diploma or lower was associated with an OR of 0.70 and showed a negative impact on consultation behavior. In this regard, considering health literacy, it is thought to be beneficial for women to receive information and educational opportunities, such as maternity classes during pregnancy. In the case of a first pregnancy and childbirth, as there is likely to be greater anxiety, we would like to emphasize the importance of this point, given that contact with professionals during pregnancy can have a significant impact on the development of trust and the recognition of sources for support, and these opportunities are particularly crucial [39].

### 5.2. Postpartum Women

The analysis of consultation behavior revealed that concerns about “children and childcare” were the strongest predictor of seeking a consultation (OR = 1.62). Postpartum abnormalities (OR = 1.23) and “physical issues” (OR = 1.28) were also significant factors. Previous studies have revealed that the main postpartum problems include “breastfeeding,” “lack of sleep,” “baby’s crying,” “caring for older children,” and “housework.” For primiparas, “breastfeeding,” “insomnia,” and “baby’s crying” are predominant, while for multiparas, “caring for older children” and “housework” are more common [9]. This finding demonstrates that maternal consultation behavior is primarily centered on child-related concerns.

In the Japanese healthcare system, postpartum women receive comprehensive care through Japan’s maternal and child health system, which includes childbirth facilities, maternal health checkups, home visits by local government authorities, and health checkup for infants and young children. These frequent monitoring opportunities enable early detection of maternal physical and mental health issues, as well as potential developmental concerns in infants. This systematic approach creates multiple avenues for women to discuss their health and childcare-related concerns.

The adverse childhood experience (ACE) component “parental mental illness” (OR = 1.61) significantly influenced maternal consultation behavior. Group comparison analyses revealed that postpartum women who engaged in consultation behavior had significantly higher Kessler Psychological Distress Scale (K6) scores, with logistic regression confirming a modest correlation between elevated K6 scores (OR = 1.02) and consultation behavior. This correlation suggests an association with maternal mental health status. Children who grow up with a parent who has a mental health problem (25%) are at an increased risk of developing (health) problems themselves [40]. Additionally, individuals experiencing mental health challenges are more likely to seek assistance when daily functioning is impaired [41]. These findings underscore the importance of considering both K6 scores and family history of mental illness when developing support interventions.

Analysis of number of persons available for consult revealed that postpartum women with 3–10 confidants (OR = 1.58) or 11 ≤ confidants (OR = 2.08) demonstrated higher rates of consultation behavior. Within these available persons to consult, husbands/partners (90.8%) and mothers (84.3%) constituted the primary persons to consult. This pattern suggests that when postpartum women have limited persons to consult (≤2 confidants), these immediate family members serve as their primary support resources. The data indicate that professional consultation becomes more accessible when postpartum women have broader support networks (≥3 confidants). Previous studies have also shown that postpartum women who are in favorable environments for childrearing—such as having an average or better standard of living and access to various types of support—tend to score higher in their willingness to make use of available support opportunities [19]. Conversely, women with restricted support networks may face barriers in accessing professional support, potentially managing challenges in isolation or exclusively within their immediate family circle.

The study population was characterized by the highest proportion of women age <34 (62.1%), a high level of education (mainly university graduates or higher), and active employment. The analysis revealed that women with high school education or lower demonstrated higher consultation behavior (OR = 1.31) compared to those with university-level education. This finding aligns with established relationships between educational attainment and socioeconomic factors, for example, income levels. In Japan, the income of women with a high school education is lower compared to those with a university degree [42]. Consequently, women from lower socioeconomic backgrounds are often classified as high-risk pregnancies in the Japanese healthcare system, warranting enhanced professional support.

The association between consultation behavior and “work-related issues” (OR = 1.19) reflects broader societal changes in Japan, particularly regarding employment conditions and work–life balance awareness. Also, postpartum women aged 34 to 39 were more likely to seek consultation compared to those under 34 (OR 1.15). Postpartum women in their 30s to 40s are often in their 10th to 15th year of service, a period marked by frequent job transfers, changes in roles, and shifts in position at the workplace. This age group is considered the generation most likely to be engaged in “double care,” simultaneously managing both childcare and eldercare [43]. It has been shown that the burden of roles in family life, such as childcare, caregiving, and housework, negatively impacts the work–life balance of working women, which in turn adversely affects their mental health [44]. To address the concerns and mental health issues of working mothers, government initiatives such as work style reforms and the promotion of paternity leave, as well as social efforts like companies creating more supportive work environments, are necessary [44].

Regarding the experience of bullying in the ACE items, a positive effect on consultation was confirmed in all groups: pregnant women (OR = 1.42), postpartum women (OR = 1.26), and primiparous women (OR = 1.54). In a study on the impact of bullying victimization on the use of mental health services from childhood to middle age, those who were bullied in childhood had a higher rate of service use compared to those who were not. Being bullied in childhood is associated with poor mental health up to middle age [45]. The fact that K6 scores were related to consultation behavior in postpartum women also showed a similar trend to previous studies. Although it is not easy to grasp the experience of bullying, it is necessary to consider it along with K6 and other ACE items as a factor leading to support.

Finally, based on the analysis results, we will further discuss the fact that a slight negative correlation was observed between consultation behavior and both the Edinburgh Postnatal Depression Scale (EPDS) score (odds ratio = 0.98) and the Mother-to-Infant Bonding Scale—Japanese version (MIBS-J) score (odds ratio = 0.98). The inverse relationship between EPDS scores and consultation behavior potentially reflects the impact of mental health stigma. Such stigma creates barriers to both depression diagnosis and treatment access, often leading individuals to avoid discussing mental health concerns and concealing symptoms to evade perceived prejudice [46]. Research indicates that women without prior mental health diagnoses face greater challenges in accessing depression care and demonstrate lower treatment utilization rates compared to those with established diagnoses. A prominent barrier is the internalized belief in self-reliance for problem resolution [47]. There is also the possibility that postpartum women may underreport their own symptoms, making close and continuous contact with healthcare professionals essential [48]. The negative association between MIBS-J scores and consultation behavior may reflect a diminished interest in child-related matters. The MIBS-J’s two-factor structure includes parental lack of affection and consideration as its primary factor [25], which may inhibit consultation behavior. Evidence suggests that among women with postpartum depression, those who sought treatment independently demonstrated lower bonding disorder scores compared to non-treatment-seeking individuals [49]. These findings underscore the importance of recognizing and addressing these behavioral patterns when developing support strategies for women with elevated EPDS or MIBS-J scores.

## 6. Implications and Limitations

In this study, we examined the consultation behaviors of pregnant and postpartum women with public institutions and the factors influencing them. As a result, the backgrounds and actual conditions of those who sought consultations and those who did not became clear.

It has been suggested that pregnant and postpartum women facing physical, mental, family, and interpersonal issues are more likely to seek consultation, and the high-risk approach within the maternal and child health system creates multiple pathways for these women to discuss concerns related to their health and childcare. First, it is strongly emphasized that for pregnant women, designing screening and preventive interventions must carefully consider whether they are primigravida or multigravida [50], and the results of this study also highlight this point, which we hope to apply in future support. Second, it is noteworthy that a negative correlation was found between EPDS scores, bonding, and consultation behavior among postpartum women. In Japan, EPDS and bonding are used as screening tools for risk assessment of postpartum depression and abuse through population-based approaches such as postpartum home visits. A more effective use of these tools remains a challenge, and it is desirable to provide training to improve the technical quality of risk assessments, as well as for professionals involved in maternal and child health to collaborate in building trusting relationships between supporters and pregnant or postpartum women, thereby facilitating access to psychological and mental health support [51].

Regarding experiences of childhood adversity, it has been shown that memories of warm interactions with caregivers mitigate the impact of childhood abuse on trauma exposure in the next generation [52]. From this, it is necessary to assess the presence or absence of adverse childhood experiences as much as possible going forward and provide support as needed. Additionally, by actively incorporating support, which includes the perspective of Protective and Compensatory Experiences (PACEs) in response to adverse experiences, it is believed that more effective assistance can be achieved.

Finland facilitates early detection, prevention, and early support for issues faced by children and families through continuous assistance, achieving high effectiveness [53,54]. There are several local governments in Japan that have adopted this system to provide support, but depending on the size of the municipality, challenges in management have also been pointed out. Moving forward, while taking into account the characteristics of each local government, I would like to consider the methods of providing support that maintain trust, based on a population approach targeting all pregnant and postpartum women, including those who are less likely to seek support on their own.

For pregnant and postpartum women to receive appropriate support, they need to develop the ability to accept and utilize that support, known as “receiving support.” This study also considers the possibility that some women were connected to support without actively seeking consultation themselves, so it was not possible to confirm whether the women themselves initiated contact for consultation. It is necessary not only for support providers to recognize the need for assistance and actively offer it but also to explore ways to enhance the self-care abilities of pregnant and postpartum women so they can seek help when needed. To achieve this, further investigation into how pregnant women who are not classified as high-risk specifically access consultation and support is required.

This study is a cross-sectional study and does not allow for longitudinal analysis. Factors influencing whether pregnant and postpartum women seek consultation are likely to change over time. Therefore, longitudinal analysis will be necessary in the future. Additionally, this study is a cross-sectional study, which precludes causal inference. Moreover, the survey data are based on self-reports, which may result in the underreporting or omission of symptoms. The data used in this study were sourced from panel members from an internet research company, but this may have introduced bias into the results. Since there are limitations in these areas, we would like to address them as future challenges.

## 7. Conclusions

This study demonstrated that the presence of physical, mental, family, and interpersonal risks influences the consultation behavior of pregnant and postpartum women in Japan. Moving forward, alongside a high-risk approach that connects at-risk pregnant and postpartum women to consultations, we aim to establish a support network from a population approach perspective that makes it easier for women without risks to seek consultations, thereby contributing to the creation of a system where all pregnant and postpartum women can access support.

## Figures and Tables

**Table 1:** Characteristics of pregnant women (N = 1380).

Variables	Category	M ± SD	M ± SD	M ± SD	x^2^/*t*-Test/ U-Test
		Total Sample n (%)	Consultation Group n (%)	Non-Consultation Group n (%)	*p*-Value
**Socio-demographic variables**					0.41
Age (years)	<34	940 (68.1)	400 (66.8)	540 (69.1)	
	34–39	381 (27.6)	169 (28.2)	212 (27.1)	
	40≤	59 (4.3)	30 (5.0)	29 (3.7)	
Education	College or higher	798 (57.8)	364 (60.8)	434 (55.6)	0.05
	High school graduate	582 (42.2)	235 (34.2)	347 (44.4)	
Occupational status	Employed	957 (69.3)	426 (71.1)	531 (68.0)	0.21
	Unemployed	423 (30.7)	173 (28.9)	250 (32.0)	
Partner annual income (million JPY)	<1.3	40 (2.9)	19 (3.2)	21 (2.7)	0.73
	1.3–5.0	637 (46.2)	281 (46.9)	356 (45.6)	
	5.0<	703 (50.9)	299 (49.9)	404 (51.7)	
Annual income (million JPY)	<1.3	544 (39.4)	226 (37.7)	318 (40.7)	0.41
	1.3–3.0	255 (18.5)	109 (18.2)	146 (18.7)	
	3.0 <	581 (42.1)	264 (44.1)	317 (40.6)	
**Physical and obstetrical variables**					
Sleep duration (h)		6.8 ± 1.45	6.7 ± 1.38	6.9 ± 1.50	0.05
Number of births (number)		0.6 ± 0.84	0.7 ± 0.89	0.5 ± 0.78	<0.01
Abnormalities during pregnancy	No	892 (64.6)	387 (64.6)	505 (64.7)	0.98
	Yes	488 (35.4)	212 (35.4)	276 (35.3)	
**Psychological variables**					
K6 (points)		7.6 ± 8.17	8.2 ± 8.27	7.1 ± 8.07	0.04
EPDS (points)		6.5 ± 5.07	6.7 ± 5.06	6.3 ± 5.07	0.09
Happiness (points)		8.2 ± 1.57	8.3 ± 1.53	8.2 ± 1.59	0.28
CCHL (points)		17.8 ± 3.45	18.0 ± 3.61	17.6 ± 3.31	0.04
**Problems**					0.84
Pregnancy and childbirth	No	428 (31.0)	184 (30.7)	244 (31.2)	
	Yes	952 (69.0)	415 (69.3)	537 (68.8)	
Economic	No	552 (40.0)	240 (40.1)	312 (39.9)	0.97
	Yes	828 (60.0)	359 (59.9)	469 (60.1)	
Physical	No	722 (52.3)	293 (48.9)	429 (54.9)	0.03
	Yes	658 (47.7)	306 (51.1)	352 (45.1)	
Family relationships	No	1081 (78.3)	462 (77.1)	619 (79.3)	0.34
	Yes	299 (21.7)	137 (22.9)	162 (20.7)	
Marital relationship	No	1080 (78.3)	457 (76.3)	623 (79.8)	0.12
	Yes	300 (21.7)	142 (23.7)	158 (20.2)	
Childcare and children	No	448 (32.5)	153 (25.5)	295 (37.8)	<0.01
	Yes	932 (67.5)	446 (74.5)	486 (62.2)	
Work	No	722 (52.3)	288 (48.1)	434 (55.6)	0.01
	Yes	658 (47.7)	311 (51.9)	347 (44.4)	
**Home environment and relationships**					
Number of household members (persons)		2.62 ± 0.91	2.7 ± 0.93	2.5 ± 0.88	<0.01
Time spent on housework and childcare (h)		5.0 ± 4.68	5.6 ± 4.98	4.6 ± 4.39	<0.01
Number of persons available for consult (persons)	≤2	446 (32.3)	179 (29.9)	267 (34.2)	0.09
	3–10	916 (66.4)	409 (68.3)	507 (64.9)	
	11≤	18 (1.3)	11 (1.8)	7 (0.9)	
Family APGAR (points)		12.6 ± 3.90	12.8 ± 3.69	12.5 ± 4.05	0.83
**ACEs**					0.97
Parent’s death	No	1329 (96.3)	577 (96.3)	752 (96.3)	
	Yes	51 (3.7)	22 (3.7)	29 (3.7)	
Divorced parents	No	1220 (88.4)	531 (88.6)	689 (88.2)	0.81
	Yes	160 (11.6)	68 (11.4)	92 (11.8)	
Parental mental illness	No	1292 (93.6)	552 (92.2)	740 (94.8)	0.05
	Yes	88 (6.4)	47 (7.8)	41 (5.2)	
Violence from father to mother	No	1290 (93.5)	552 (92.2)	738 (94.5)	0.08
	Yes	90 (6.5)	47 (7.8)	43 (5.5)	
Physical abuse	No	1317 (95.4)	561 (93.7)	756 (96.8)	<0.01
	Yes	63 (4.6)	38 (6.3)	25 (3.2)	
Neglect	No	1360 (98.6)	590 (98.5)	770 (98.6)	0.89
	Yes	20 (1.4)	9 (1.5)	11 (1.4)	
Emotional abuse	No	1190 (86.2)	497 (83.0)	693 (88.7)	<0.01
	Yes	190 (13.8)	102 (17.0)	88 (11.3)	
Economic poverty	No	1233 (89.3)	524 (87.5)	709 (90.8)	0.05
	Yes	147 (10.7)	75 (12.5)	72 (9.2)	
Bullying	No	1040 (75.4)	419 (69.9)	621 (79.5)	<0.01
	Yes	340 (24.6)	180 (30.1)	160 (20.5)	
Sexual victimization	No	1331 (96.4)	571 (95.3)	760 (97.3)	0.05
	Yes	49 (3.6)	28 (4.7)	21 (2.7)	

Note: M, Mean. SD, Standard Deviation. EPDS, Edinburgh Postpartum Depression Scale. ACEs, Adverse Childhood Experiences. CCHL, Communicative and Critical Health Literacy. Family APGAR, Adaptability, Partnership, Growth, Affection, and Resolve.

**Table 2:** Characteristics of postpartum women (N = 4847).

Variables	Category	M ± SD	M ± SD	M ± SD	x^2^/*t*-Test/ U-Test
		Total Sample n (%)	Consultation Group n (%)	Non-Consultation Group n (%)	*p*-Value
Socio-demographic variables					0.06
Age (years)	<34	3010 (62.1)	1615 (60.9)	1395 (63.6)	
	34–39	1607 (33.2)	917 (34.6)	690 (31.4)	
	40≤	230 (4.7)	110 (5.0)	527 (24.0)	
Education	College or higher	2984 (52.5)	1738 (55.8)	1246 (48.4)	<0.01
	High school graduate	2704 (47.5)	1377 (44.2)	1327 (51.6)	
Occupational status	Employed	3259 (67.2)	1824 (68.8)	1435 (65.4)	0.01
	Unemployed	1588 (32.8)	828 (32.8)	760 (34.6)	
Partner annual income (million JPY)	<1.3	170 (3.5)	93 (3.5)	77 (3.5)	0.87
	1.3–5.0	2177 (44.9)	1182 (44.6)	995 (45.3)	
	5.0<	2500 (51.6)	1377 (51.9)	1123 (51.2)	
Annual income (million JPY)	<1.3	2285 (47.1)	1216 (45.9)	1069 (48.7)	0.14
	1.3–3.0	961 (19.8)	535 (20.2)	426 (19.4)	
	3.0 <	1601 (33.0)	901 (34.0)	700 (31.9)	
**Physical and obstetrical variables**					
Sleep duration (h)		6.3 ± 1.24	6.3 ± 1.23	6.3 ± 1.268	0.87
Number of births (number)		1.5 ± 0.90	1.5 ± 1.02	1.6 ± 0.745	0.07
Types of birth	Vaginal delivery	3926 (81.0)	2137 (80.6)	1789 (81.5)	0.42
	Cesarean section	921 (19.0)	515 (19.4)	406 (18.5)	
Gestational age at childbirth (week)		38.7 ± 1.96	38.7 ± 1.90	38.7 ± 2.04	0.58
Birth weight (g)		3010 ± 413	3012± 414	3008 ± 411	0.75
Abnormalities during pregnancy	No	3750 (65.9)	2046 (65.7)	1704 (66.2)	0.67
	Yes	1938 (34.1)	1069 (34.3)	869 (33.8)	
Postpartum anomalies	No	4697 (82.6)	2511 (80.6)	2186 (85.0)	<0.01
	Yes	991 (17.4)	604 (19.4)	387 (15.0)	
**Psychological variables**					
K6 scale (points)		8.1 ± 8.40	8.4 ± 8.35	7.8 ± 8.45	<0.01
EPDS (points)		5.9 ± 4.99	5.8 ± 4.80	6.1 ± 5.21	0.62
MIBS-J (points)		3.0 ± 3.60	2.8 ± 3.30	3.2 ± 3.91	0.56
Happiness (points)		8.26 ± 1.59	8.3 ± 1.52	8.1 ± 1.67	0.02
CCHL (points)		17.6 ± 3.60	18.0 ± 3.49	17.2 ± 3.70	<0.01
**Problems**					
Pregnancy and childbirth	No	3838 (79.2)	2040 (76.9)	1798 (81.9)	<0.01
	Yes	1009 (20.8)	612 (23.1)	397 (18.1)	
Economic	No	2292 (47.3)	1167 (44.0)	1125 (51.3)	<0.01
	Yes	2555 (52.7)	1485 (56.0)	1070 (48.7)	
Physical	No	2898 (59.7)	1451 (54.7)	1443 (65.7)	<0.01
	Yes	1953 (40.3)	1201 (45.3)	752 (34.3)	
Family relationships	No	3907 (80.6)	2100 (79.2)	1807 (82.3)	<0.01
	Yes	940 (19.4)	552 (20.8)	388 (17.7)	
Marital relationship	No	3472 (71.6)	1837 (69.3)	1635 (74.5)	<0.01
	Yes	1375 (28.4)	815 (28.4)	560 (25.5)	
Childcare and children	No	2018 (41.6)	929 (35.0)	1089 (49.6)	<0.01
	Yes	2829 (58.4)	1723 (65.0)	1106 (50.4)	
Work	No	2340 (48.3)	1138 (42.9)	1202 (54.8)	<0.01
	Yes	2507 (51.7)	1514 (51.7)	993 (45.2)	
**Home environment and relationships**					
Number of household members (persons)		3.6 ± 0.87	3.6 ± 0.86	3.6 ± 0.88	0.11
Time spent on housework and childcare (hours)		11.1 ± 5.60	11.1 ± 5.57	11.0 ± 5.65	0.51
Family APGAR (points)		11.9 ± 4.22	12.0 ± 4.09	11.7 ± 4.38	0.07
Number of persons available for consult (persons)	≤2	1874 (38.7)	898 (33.9)	976 (44.5)	<0.01
	3–10	2906 (60.0)	1711 (64.5)	1195 (54.4)	
	11≤	67 (1.4)	43 (1.6)	24 (1.1)	
**Someone to consult with**					
Husband	Yes	4337 (90.8)	2367 (90.6)	1970 (91.0)	0.57
	No	441 (9.2)	247 (9.4)	194 (9.0)	
Mother	Yes	4027 (84.3)	2216 (84.8)	1811 (83.7)	0.30
	No	751 (15.7)	398 (15.2)	353 (16.3)	
Own siblings	Yes	2561 (53.6)	1369 (52.4)	1192 (55.1)	0.06
	No	2217 (46.4)	1245 (47.6)	972 (44.9)	
**ACEs**					
Parent’s death	No	4623 (95.4)	2526 (95.2)	2097 (95.5)	0.64
	Yes	224 (4.6)	126 (4.8)	98 (4.5)	
Divorced parents	No	4243 (87.5)	2335 (88.0)	1908 (86.9)	0.24
	Yes	604 (12.5)	317 (12.0)	287 (13.1)	
Parental mental illness	No	4554 (94.0)	2461 (92.8)	2093 (95.4)	<0.01
	Yes	293 (6.0)	191 (7.2)	102 (4.6)	
Violence from father to mother	No	4478 (92.4)	2444 (92.2)	2034 (92.7)	0.51
	Yes	369 (7.6)	208 (7.8)	161 (7.3)	
Physical abuse	No	4606 (95.0)	2512 (94.7)	2094 (95.4)	0.28
	Yes	241 (5.0)	140 (5.3)	101 (4.6)	
Neglect	No	4769 (98.4)	2607 (98.3)	2162 (98.5)	0.59
	Yes	78 (1.6)	45 (1.7)	33 (1.5)	
Emotional abuse	No	4052 (83.6)	2180 (82.2)	1872 (85.3)	<0.01
	Yes	795 (16.4)	472 (17.8)	323 (14.7)	
Economic poverty	No	4208 (86.8)	2289 (86.3)	1919 (87.4)	0.25
	Yes	639 (13.2)	363 (13.7)	276 (12.6)	
Bullying	No	3539 (73.0)	1856 (70.0)	1683 (76.7)	<0.01
	Yes	1308 (27.0)	796 (30.0)	512 (23.3)	
Sexual victimization	No	4633 (95.6)	2517 (94.9)	2116 (96.4)	0.01
	Yes	214 (4.4)	135 (5.1)	79 (3.6)	

Note: M, Mean. SD, Standard Deviation. EPDS, Edinburgh Postpartum Depression Scale. Bonding, Mother-to-Infant Bonding Scale (MIBS)-J. ACEs, Adverse Childhood Experiences. CCHL, Communicative and Critical Health Literacy. Happiness, 1–10 points; the higher the score, the happier they are. Family APGAR, Adaptability, Partnership, Growth, Affection, and Resolve.

**Table 3:** Logistic regression analysis of pregnant and postpartum women who consulted public institutions.

		Pregnant Women		Postpartum Women	
Variables		OR (95%CI)	*p*-Value	OR (95%CI)	*p*-Value
**Socio-demographic variables**					
Age (years)					
<34					
34–39		1.07 (0.82–1.39)	0.61	1.15 (1.01–1.31)	0.04
40≤		1.48 (0.84–2.61)	0.17	1.09 (0.81–1.45)	0.58
Education					
College or higher					
High school graduate or lower		0.79 (0.62–1.01)	0.06	1.31 (1.15–1.48)	<0.01
Occupation					
Unemployed					0.22
Employed		1.07 (0.78–1.47)	0.67	0.90 (0.76–1.07)	
Partner annual income (million JPY)					
<1.3					
1.3–5.0		1.51 (0.76–3.00)	0.25	1.16 (0.83–1.63)	0.37
5.0<		1.16 (0.91–1.48)	0.24	1.00 (0.88–1.13)	0.97
Annual income (million JPY)					
<1.3			0.69		0.59
1.3–3.0		0.87 (0.62–1.20)	0.39	1.09 (0.91–1.30)	0.36
3.0<		0.95 (0.69–1.31)	0.75	1.07 (0.90–1.27)	0.43
**Physical and obstetrical variables**					
Sleep duration (h)		1.00 (0.92–1.09)	0.97	1.03 (0.98–1.08)	0.23
Number of births		1.40 (1.11–1.77)	0.01	1.03 (0.94–1.13)	0.58
Types of birth					
Vaginal delivery					
Cesarean section				1.01 (0.87–1.19)	0.86
Gestational age at childbirth (week)				1.02 (0.98–1.05)	0.38
Birth weight (g)				1.00 (1.00–1.00)	0.77
Abnormalities during pregnancy	(Yes)	1.01 (0.80–1.28)	0.93	1.02 (0.90–1.15)	0.78
Postpartum abnormalities	(Yes)			1.23 (1.04–1.45)	0.01
**Psychological Variables**					
K6		1.02 (1.00–1.04)	0.13	1.02 (1.01–1.03)	<0.01
EPDS		1.00 (0.96–1.03)	0.87	0.98 (0.96–1.00)	0.02
MIBS-J				0.98 (0.96–1.00)	0.02
Happiness		1.06 (0.97–1.17)	0.22	1.09 (1.04–1.14)	<0.01
CCHL		1.02 (0.99–1.06)	0.16	1.05 (1.03–1.06)	<0.01
Problems					
Pregnancy and childbirth (Yes)		1.04 (0.77–1.41)	0.79	1.03 (0.88–1.21)	0.68
Physical (Yes)		1.06 (0.81–1.37)	0.68	1.28 (1.12–1.47)	<0.01
Economic (Yes)		0.83 (0.63–1.10)	0.19	1.14(0.99–1.31)	0.08
Family relationships (Yes)		1.00 (0.73–1.39)	0.98	0.93 (0.78–1.11)	0.41
Marital relationship (Yes)		0.98 (0.71–1.36)	0.90	1.14 (0.97–1.33)	0.11
Childcare and children (Yes)		1.81 (1.37–2.39)	<0.01	1.62 (1.42–1.86)	<0.01
Work (Yes)		1.18 (0.92–1.53)	0.20	1.19 (1.04–1.37)	0.01
**Home environment and relationships**					
Number of household members (persons)		0.92 (0.75–1.14)	0.45	0.97 (0.88–1.06)	0.48
Time spent on housework and childcare		1.04 (1.01–1.07)	0.016	1.00 (0.99–1.01)	0.45
Number of persons available for consult					
≤2		1.27 (0.97–1.66)	0.08	1.58 (1.39–1.80)	<0.01
3–10		2.18 (0.79–6.00)	0.13	2.08 (1.22–3.55)	<0.01
11≤		1.03 (0.99–1.07)	0.14	1.00 (0.99–1.02)	0.67
Family APGAR (points)					
ACEs					
Parent’s death (Yes)		0.84 (0.46–1.56)	0.59	1.00 (0.75–1.33)	0.98
Divorced parents (Yes)		0.74 (0.50–1.10)	0.13	0.90 (0.74–1.09)	0.27
Parental mental illness (Yes)		1.33 (0.82–2.16)	0.25	1.61 (1.23–2.11)	<0.01
Violence from father to mother (Yes)		1.13 (0.68–1.87)	0.63	0.99 (0.77–1.27)	0.95
Physical abuse (Yes)		1.53 (0.84–2.80)	0.17	0.98 (0.71–1.35)	0.90
Neglect (Yes)		0.90 (0.34–2.38)	0.83	0.88 (0.53–1.46)	0.63
Emotional abuse (Yes)		1.19 (0.82–1.74)	0.36	1.10 (0.91–1.34)	0.32
Economic poverty (Yes)		1.26 (0.85–1.88)	0.26	1.03 (0.85–1.26)	0.74
Bullying (Yes)		1.42 (1.07–1.88)	0.02	1.26 (1.09–1.46)	<0.01

**Table 4:** Logistic regression analysis of primigravidaewho consulted public institutions.

Variables		OR (95%CI)	*p*-Value
**Socio-demographic variables**			
Age (years)			
<34			
34–39		1.13 (0.75–1.70)	0.56
40≤		2.94 (1.19–7.28)	0.02
Education			
College or higher			
High school graduate or lower		0.70 (0.49–0.99)	0.04
Occupation			
Unemployed			
Employed		1.29 (0.83–1.98)	0.26
Partner annual income (million JPY)			
<1.3			
1.3–5.0		1.39 (0.52–3.67)	0.51
5.0<		1.13 (0.79–1.61)	0.50
Annual income (million JPY)			
<1.3			
1.3–3.0		1.17 (0.74–1.85)	0.49
3.0<		1.13 (1.03–1.25)	0.45
**Physical and obstetrical variables**			
Sleep duration (h)		1.00 (0.88–1.12)	0.93
Abnormalities during pregnancy	(Yes)	0.93 (0.67–1.30)	0.68
**Psychological variables**			
K6		1.01 (0.98–1.04)	0.54
EPDS		1.02 (0.97–1.07)	0.38
Happiness		1.07 (0.94–1.22)	0.34
CCHL		1.00 (0.95–1.05)	0.96
Problems			
Pregnancy and childbirth (Yes)		1.31 (0.78–2.20)	0.31
Physical (Yes)		1.27 (0.89–1.83)	0.19
Economic (Yes)		0.79 (0.53–1.19)	0.26
Family relationships (Yes)		0.92 (0.59–1.43)	0.70
Marital relationship (Yes)		0.86 (0.54–1.37)	0.53
Childcare and children (Yes)		1.47 (0.98–2.22)	0.07
Work (Yes)		1.23 (0.85–1.77)	0.27
**Home environment and relationships**			
Time spent on housework and childcare		1.80 (1.04–3.11)	<0.01
ACEs			
Parent’s death (Yes)		0.57 (0.24–1.34)	0.19
Divorced parents (Yes)		0.58 (0.34–1.01)	0.06
Parental mental illness (Yes)		1.15 (0.59–2.24)	0.68
Violence from father to			
mother (Yes)		0.99 (0.48–2.04)	0.98
Physical abuse (Yes)		0.77 (0.31–1.93)	0.58
Neglect (Yes)		0.49 (0.10–2.49)	0.39
Emotional abuse (Yes)		1.81 (1.05–3.12)	0.03
Economic poverty (Yes)		1.06 (0.60–1.89)	0.86
Bullying (Yes)		1.54 (1.03–2.30)	0.04
Sexual victimization (Yes)		1.71 (0.76–3.82)	0.20

Note: OR, odds ratio; 95% CI, 95% confidence interval.

## Data Availability

The data sets presented in this article are not readily available ethical considerations regarding data sharing.

## Supplementary Materials

The following supporting information can be downloaded at: https://www.mdpi.com/article/10.3390/healthcare13121422/s1, Table S1: Characteristics of primigravidae (N = 725).
