# Supplementary material for: Influence on Consultation Behavior of Pregnant and Postpartum Women in Japan: Insights from a Nation-Wide Survey
Source: Healthcare (Basel). 2025 Jun 13;13(12):1422. doi: 10.3390/healthcare13121422 (PMC12192908; doi:10.3390/healthcare13121422)
Supplement: Supplementary file 1 [file healthcare-13-01422-s001.zip › healthcare-3666297-supplementary.pdf]

Table S1. Characteristics of primigravidae (N=725)

Variables	Category	*M ± SD* Total sample n (%)	*M ± SD* Consulting group n (%)	*M ± SD* No consultation group n (%)	*x^2/t-Test/ U-test* *p-value*
**Socio-Demographic variables**					
Age (Years)	<34	555 (76.6)	209 (74.4)	346 (77.9)	*0.08*
	34-39	145 (20.0)	57 (20.3)	88 (19.8)	
	40≤	25 (3.4)	15 (5.3)	10 (2.3)	
Education	College or higher	437 (60.3)	183 (65.1)	254 (57.2)	***0.03***
	High school graduate	288 (39.7)	98 (34.9)	190 (42.8)	
Occupation	Employed	513 (70.8)	202 (71.9)	311 (70.0)	*0.60*
	Un Employed	212 (29.2)	79 (28.1)	133 (30.0)	
Partner annual income (million yen)	< 1.3	21 (2.9)	9 (3.2)	12 (2.7)	*0.91*
	1.3 - 5.0	349 (48.1)	136 (48.4)	213 (48.0)	
	5.0 <	355 (49.0)	136 (48.4)	219 (49.3)	
Annual income (million yen)	< 1.3	238 (32.8)	94 (33.5)	144 (32.4)	*0.60*
	1.3 - 3.0	148 (20.4)	52 (18.5)	96 (21.6)	
	3.0 <	339 (46.8)	135 (48.0)	204 (45.9)	
**Physical and Obstetrical Variables**					
Sleep duration (hour)		7.1 ± 1.38	7.1 ± 1.23	7.1 ± 1.47	*0.59*
Abnormalities during pregnancy	No	568 (65.4)	229 (67.2)	339 (64.3)	*0.39*
	Yes	300 (34.6)	112 (32.8)	188 (35.7)	
**Psychological Variables**					
K6 (point)		7.7 ± 8.07	8.7 ± 8.38	7.1 ± 7.82	*0.09*
EPDS (point)		6.6 ± 4.92	7.0 ± 5.08	6.3 ± 4.80	*0.77*
Happiness (point)		8.3 ± 1.54	8.3 ± 1.51	8.3 ± 1.55	*0.97*
CCHL (point)		17.9 ± 3.40	18.0 ± 3.70	17.8 ± 3.20	*0.36*
Problem					
Pregnancy and childbirth	No	123 (17.0)	36 (12.8)	87 (19.6)	***0.02***
	Yes	602 (83.0)	245 (87.2)	357 (80.4)	
Economic	No	258 (35.6)	100 (35.6)	158 (35.6)	*1.00*
	Yes	467 (64.4)	181 (64.4)	286 (64.4)	
Physical	No	360 (49.7)	122 (43.4)	238 (53.6)	***0.01***
	Yes	365 (50.3)	159 (56.6)	206 (46.4)	
Family relationships	No	573 (79.0)	219 (77.9)	354 (79.7)	*0.56*
	Yes	152 (21.0)	62 (22.1)	90 (20.3)	
Marital relationship	No	567 (78.2)	214 (76.2)	353 (79.5)	*0.29*
	Yes	158 (21.8)	67 (23.8)	91 (20.5)	
Childcare and children	No	222 (30.6)	68 (24.2)	154 (34.7)	***<0.01***
	Yes	503 (69.4)	213 (75.8)	290 (65.3)	
Work	No	338 (46.6)	116 (41.3)	222 (50.0)	***0.02***
	Yes	387 (53.4)	165 (58.7)	222 (50.0)	
**Home Environment and Relationships**					
Number of households (person)		2.06 ± 0.43	2.0 ± 0.41	2.0 ± 0.45	*0.94*
Housework and Childcare time (hour)		2.74 ± 1.74	2.9 ± 1.89	2.5 ± 1.63	***<0.01***
Number of persons available for consult (person)	2≤	232 (32.0)	86 (30.6)	146 (32.9)	*0.68*
	3-10	485 (66.9)	191 (68.0)	294 (66.2)	
	11≤	8 (1.1)	4 (1.4)	4 (0.9)	
Family APGAR (point)		12.9 ± 3.69	12.9 ± 3.75	13.0 ± 3.66	*0.89*
ACEs					
Parent's death	No	695 (95.9)	272 (96.8)	423 (95.3)	*0.32*
	Yes	30 (4.1)	9 (3.2)	21 (4.7)	
Divorced parents	No	640 (88.3)	255 (90.7)	385 (86.7)	*0.10*
	Yes	85 (11.7)	26 (9.3)	59 (13.3)	
Parental mental illness	No	676 (93.2)	260 (92.5)	416 (93.7)	*0.54*
	Yes	49 (6.8)	21 (7.5)	28 (6.3)	
Violence from father to mother	No	679 (93.7)	262 (93.2)	417 (93.9)	*0.71*
	Yes	46 (6.3)	19 (6.8)	27 (6.1)	

Physical abuse	No	694 (95.7)	266 (94.7)	428 (96.4)	*0.26*
	Yes	31 (4.3)	15 (5.3)	16 (3.6)	
Neglect	No	715 (98.6)	278 (98.9)	437 (98.4)	*0.57*
	Yes	10 (1.4)	3 (1.1)	7 (1.6)	
Emotional abuse	No	632 (87.2)	231 (82.2)	401 (90.3)	***<0.01***
	Yes	93 (12.8)	50 (17.8)	43 (9.7)	
Economic poverty	No	651 (89.8)	249 (88.6)	402 (90.5)	*0.40*
	Yes	74 (10.2)	32 (11.4)	42 (9.5)	
Bullying	No	546 (75.3)	193 (68.7)	353 (79.5)	***<0.01***
	Yes	179 (24.7)	88 (31.3)	91 (20.5)	
Sexual victimization	No	695 (95.9)	264 (94.0)	431 (97.1)	***0.04***
	Yes	30 (4.1)	17 (6.0)	13 (2.9)	

Note: M; Mean. SD; Standard Deviation. EPDS ; Edinburgh Postpartum Depression Scale.
ACEs; Adverse Childhood Experiences. CCHL; Communicative and Critical Health Literacy.
Family APGAR; Adaptability, Partnership, Growth, Affection, and Resolve.
